# Neurological infection and complications of SARS-CoV-2: A review

**DOI:** 10.1097/MD.0000000000030284

**Published:** 2023-02-03

**Authors:** Santosh Singh, Nikita Meher, Arifullah Mohammed, Mohammad Khairul Azhar Abdul Razab, L.V.K.S. Bhaskar, Norazlina Mat Nawi

**Affiliations:** a Biochemistry and Molecular Biology Laboratory, Department of Zoology, Guru Ghasidas Vishwavidyalaya (a Central University), Bilaspur, Chhattisgarh, India; b Department of Agriculture Science, Faculty of Agro-Based Industry, Universiti Malaysia Kelantan, Jeli, Kelantan, Malaysia; c Medical Radiation Programme, School of Health Sciences, Universiti Sains Malaysia, Health Campus, Kubang Kerian, Kelantan, Malaysia; d Department of Nuclear Medicine, Radiotherapy & Oncology, School of Medical Sciences, Universiti Sains Malaysia, Health Campus, Kubang Kerian, Kelantan, Malaysia.

**Keywords:** COVID-19, encephalopathy, neurological manifestations, SARS-CoV-2

## Abstract

The primary target of severe acute respiratory syndrome coronavirus 2 is the respiratory system including the nose and lungs, however, it can also damage the kidneys, cardiovascular system and gastrointestinal system. Many recent reports suggested that severe acute respiratory syndrome coronavirus 2 infections can also affect the central nervous system as well as peripheral nervous system that lead to the several neurological complications. The virus can break the blood brain barrier and enters the brain via haematological route or directly by the angiotensin-converting enzyme 2 receptors present on endothelial cells of many cerebral tissues. The neurological complications are manifested by headache, dizziness, encephalopathy, encephalitis, cerebrovascular disease, anosmia, hypogeusia, muscle damage, etc. This review article described the possible routes and mechanism of nervous system infection and the range of neurological complications of COVID-19 that may help the medical practitioners and researchers to improve the clinical treatment and reduce the mortality rate among patients with viral diseases.

## 1. Introduction

There was a sudden rise in the cases of pneumonia of undetermined origin in mid-December 2019 in Wuhan, Hubei province of China.^[1]^ This new infection was reported to world health organization on December 31, 2019.^[2]^ Initially it originated in the seafood wholesale market in Wuhan, Hubei province in China and known that early cases arise among those who had direct contact history with the seafood wholesale market and transmitted largely from human-to-human contact Worldwide.^[1]^ Genetic sequencing and reverse transcription polymerase chain reaction (RT-PCR) test recognized a novel human coronavirus at the lower respiratory tract of the pneumonia patients that was different from human Middle East respiratory syndrome coronavirus (MERS-CoV) and severe acute respiratory syndrome coronavirus-1 (SARS-CoV-1).^[2]^ Initially this new virus strain was named as “Wuhan coronavirus” or “2019-CoV” that was officially called as “severe acute respiratory syndrome corona virus 2 (SARS-CoV-2)” on February 11, 2020.^[1,3]^ The coronavirus is not new, it was first recognized and isolated from Chicken in 1937.^[4,5]^ Till date 7 types of coronaviruses have been identified that can infect human beings. The COVID-19 has spread Worldwide at speed like lightening because the main routes of transmission among human population are the respiratory droplets and close contact with infected people.^[6]^ world health organization declared a public health emergency to this situation that was the most severe and largest pandemic after the 1918 influenza pandemic.^[4,7]^

The primary target of this virus is respiratory system along with nose and lung, but it can also affect other vital organs like the heart, blood vessels, kidney, gut and brain. Regular symptoms of infection include fever, dry cough, breathing difficulties, fatigue, severe pneumonia and cardio-respiratory disease that ultimately leads to respiratory or lung diseases. However, recent research provokes neurological complications and symptoms that include headache, dizziness, encephalopathy, encephalitis, acute disseminated encephalomyelitis (ADEM), acute necrotizing encephalopathy (ANE), myelitis, anosmia, ageusia etc. It can affect both the central and peripheral nervous system. The first case of viral encephalitis in COVID-19 patients was reported in the Beijing Dital Hospital in early March 2020. Later on, another case was reported in Japan, where RT-PCR result of nasal swab found negative but the SARS-CoV-2 was detected in the cerebrospinal fluid (CSF) of patients, suggested both direct and indirect routes of invasion of the virus in to the brain that led the several neurological complications.^[8–10]^

## 2. Genome structure and proteins of SARS-CoV-2

Genotypically and serologically, coronaviruses are classified into 4 genera, alpha, beta, gamma and delta coronavirus.^[6]^ Before SARS- CoV-2 another 6 types of human coronaviruses (H- Coronavirus) were identified, those are SARS- CoV-1, MERS-CoV, HCoV-OC43, HCoV-229E, HCoV-NL-63, and HCoV-HKU-1^[2]^ and SARS-CoV-2, the 7th virus of this family.^[11]^ Coronaviruses are positive sense single stranded enveloped ribonucleic acid (RNA) viruses of about 100 to 150 nm in diameter,^[2]^ spherical, and oval consisting of 26 to 32 kb size genome^[12]^ that is the largest genome among all known RNA viruses^[3]^ along with a 5’ cap and 3’ poly A tail.^[13]^ SARS-CoV-2 has 29,903 bases long genome evolved earlier than the end of October 2019. It shares 96% similarity with bat Coronavirus, which reveals that bat is the intermediate host for transmitting to other animals.^[2,14,15]^

## 3. General symptoms and clinical manifestations of covid-19

The novel coronavirus causes various disorders in the animals, like infectious inflammation of the peritoneum in cats, pneumonitis and sialo dacryoadenitis in rats, inflammation of liver and brain in mice, inflammation of the intestine in dogs and calves, inflammation of the gastrointestinal tract and brain in young piglets, inflammation of the mucous membrane in the bronchial tubes and nephrosis in chickens.^[2]^ Study based on Chinese center for disease control, among all SARS-CoV-2 infected people, 80.9% person does not develop any symptoms or developed only mild pneumonia, but they excrete numerous viruses at the initial stage of infection.^[2,16–18]^ People suffering from comorbidities, having cardiac and respiratory disorders, hypertension and diabetes, cardiovascular and lung diseases, sickle cell disease, cancer, kidney disease, pregnant women being a cigarette smoker (NIH, 2021), showed highest mortality rate.^[2,18]^ Nevertheless, there were no effective drugs or vaccines available to fight against these viruses, only early diagnosis and isolation of the infected people play major role to control this pandemic.^[19]^

## 4. Knowledge from other viruses infecting the nervous system

**Figure 1.**
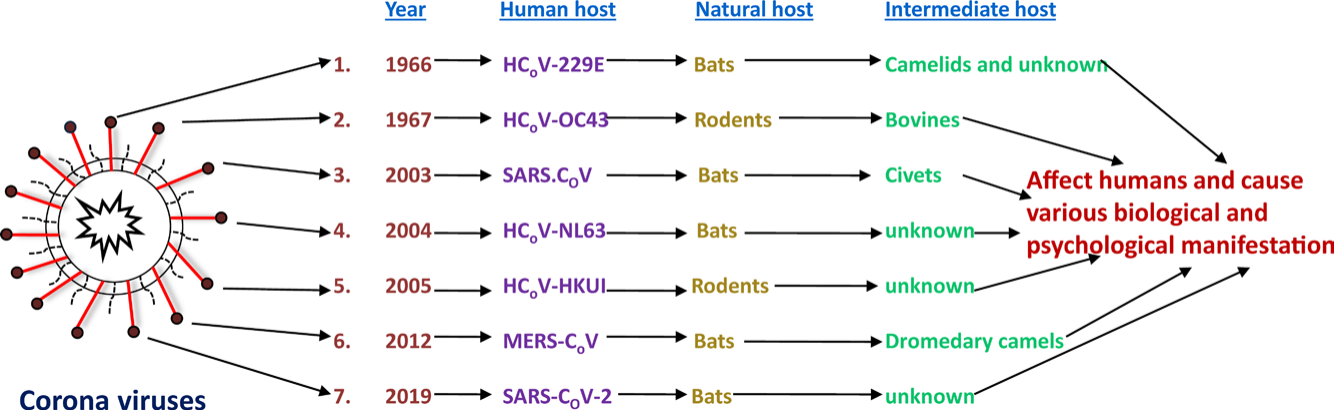
Other viruses infecting the nervous system. SARS-CoV-1, MERS-CoV and SARS-CoV-2 are well known to infect the nervous system and cause various neurological manifestations. MERS-CoV = middle east respiratory syndrome corona virus, SARS-CoV-1 = severe acute respiratory syndrome corona virus 1, SARS-CoV-2 = severe acute respiratory syndrome corona virus 2.

Many viral infections damaged the structure and functions of nervous system. Though, blood brain barrier (BBB) and CSF provide protection to the central nervous system (CNS), several viruses can break the BBB and invade the brain by different mechanism, paracellularly, intracellularly or by “Trojan horse” pathway. In a Trojan horse pathway, some viruses use infected blood stem cells to enter the CNS through the blood supply.^[20]^ When the virus infects CNS, it causes inflammation of the brain. Similarly, systemic virus infection leads toxic encephalopathy and ADEM by damaging the protective covering of nerve fibers^[8]^ that lead to BBB dysfunction, increased permeability and increase in number of WBC in CSF. Examples of such viruses that infect the brain are human immuno deficiency virus type-I, rhabdo virus, flavi virus, mouse adenovirus type-I, herpes simplex virus, influenza virus, parainfluenza virus, cytomegalovirus, lymphocytic choriomeningitis virus, arbovirus, mumps virus, parvo virus B19, measles virus, human T- cell leukaemia virus, enterovirus, bunya virus, etc. Some viruses infect the microglia, astrocytes and macrophages of CNS.^[8,21]^ Figure 1 has mentioned some viruses infecting the nervous system. (Fig. 1).

The virus can enter CNS through hematogenous route. Viruses like measles and mumps enter through respiratory system, human enteroviruses enter through gastrointestinal tract and cause oropharyngeal and lymphoid tissue infection. The arbovirus enter skin by insect bite and taken to the lymph node by Langerhans cells. Thereafter, from secondary lymphoid tissue, viruses released into blood stream and leads to many infections. Some viruses cross the BBB by directly invading the vascular endothelial cells and enter into brain, while others invade the circumventricular organs, which provide a special type of connection between the CNS and peripheral blood and choroid plexus. The second way of viral entry inside the brain is the peripheral nerves. The rabies virus and the polio virus infect the brain by using peripheral motor neurons. Rabies virus, herpes simplex virus-1, Nipah virus and influenza virus enter CNS through olfactory nerves.^[20]^

### 4.1. SARS-CoV-1

Inflammation of the brain, cerebrovascular accident and polyneuropathy had developed in patients with SARS.^[8]^ Autopsy study reveals the sign of swelling of the brain and meningeal vasodilation. Ischemic changes of neurons, damage to the myelin sheath that covers the nerve fibers in human brain, optic nerves and spinal cord, infiltration of immune cells such as monocytes and lymphocytes in the vessel walls as well as SARS-CoV virus detected in the brain.^[8]^

### 4.2. MERS-CoV

A retrospective study shows that insanity had developed in 25.7% and seizure 8.6% in MERS.^[8]^ Neurological symptoms including Guillain-Barre-syndrome (GBS), cerebrovascular accident, unconsciousness, paralysis had developed in 1/5 patients with MERS-CoV infection but all these symptoms were developed after 2 to 3 weeks, not concomitantly with respiratory symptoms.^[8]^ Many studies reported that patients of encephalopathy with seizure infected with SARS-CoV-1 in the CSF. They are also had neuromuscular disease like myopathy and neuropathy. Similarly, patients with MERS may have Bickerstaff’s brainstem encephalitis, cerebrovascular disease and encephalomyelitis.^[4]^

## 5. Routes of infection and pathogenic mechanism of nervous system infection by SARS-CoV-2

**Figure 2.**
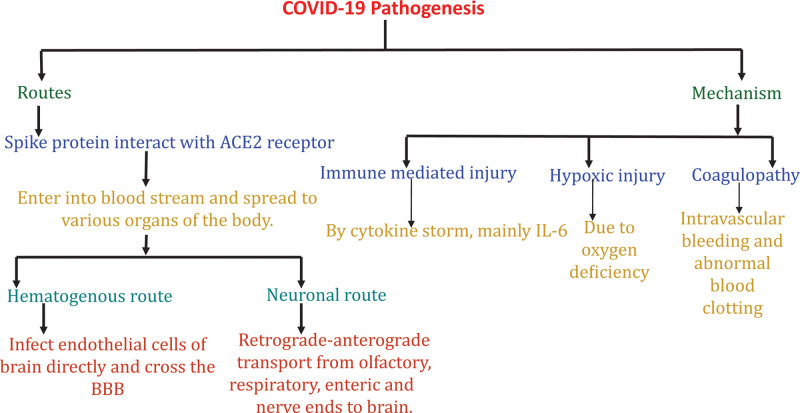
Routes of infection and pathogenic mechanisms of Nervous system infection by SARS-COV-2. Hematogenous and neuronal routes are the main routes by which SARS-CoV-2 infect human brain. The receptor of this virus is ACE-2 receptor and the mechanism by which it causes neurological manifestations are cytokine storm caused by immune mediated injury, hypoxic injury and coagulopathy. SARS-CoV-2 = severe acute respiratory syndrome corona virus 2.

The neurotropic virus like SARS-CoV-2 invade the brain directly from cribriform plate, which is situated close to the olfactory bulb.^[9,11]^ The anatomical organization of olfactory nerves is unique, the olfactory bulb is not protected by dura, so it creates a connection between the nasal cavity and the brain.^[4,11,22]^ This evidence is proved by anosmia and ageusia in the patients of COVID-19.^[9,11]^ CNS may directly affect through hematogenous route also.^[6]^ Presence of viral proteins and RNA in CSF confirm the direct invasion of virus into the nervous system.^[3,8]^ Figure 2 has highlighted the routes of infection and neurological pathogenesis of SARS-CoV-2 (Fig. 2)

### 5.1. Haematogenous route

The virus excretes and circulate through the blood and bind with its receptor wherever it is expressed. The receptor for the spike protein of SARS-CoV-2 is angiotensin converting enzyme 2 (ACE2) which is expressed in many organs all over the body and causes various manifestations.^[11,23]^ This receptor is also expressed in various cells and tissues of brain, which make the virus to infect the brain.^[11]^ ACE2 receptor negatively regulate the renin – angiotensin system.^[6]^ The ACE2 receptors are highly expressed in the CNS and peripheral nervous system (PNS) with severe neurological complications.^[24,25]^ The enzyme ACE increase the concentration of angiotensin II, a vasoconstrictor in the body and act antagonistic to ACE2. ACE2 is responsible for the breakdown and conversion of angiotensin II into angiotensin, a vasodilator. So ACE2 lowers the blood pressure by decreasing vasoconstrictors and increasing the vasodilators.^[1]^ Because ACE2 receptors regulate the blood pressure, blockage of ACE2 by SARS-CoV-2 at the time of entry inside cells leads to its depletion and angiotensin II are accumulated which would result in water and sodium retention and vasoconstriction, which ultimately increase the blood pressure. High angiotensin II concentration also promote blood coagulation and inflammation.^[6,25]^ Some patients developed unusual high blood pressure and increased risk of cerebrovascular disease when ACE2 expression is decreased by binding with virus spike proteins.^[6]^

A recent study reveals that the binding capacity of spike protein to ACE2 receptor in SARS- CoV-2 are 10 to 20 times more than the spike protein of SARS-CoV-1.^[11,26]^ In the micro-vessels of brain, basigin and neuropilin 1 (NRP1) are more in number than ACE2, which suggest that SARS-CoV-2 need these basigin and NRP1 receptors to invade the CNS.^[25]^ NRP1 express in the olfactory epithelium and both NRP1 and ACE2 may be involved in transmission of SARS-CoV-2 from olfactory epithelium to brain.^[6]^ More recent studies also reveals that the (transmembrane serine protease 2 and transmembrane serine protease 4) act as coreceptor for ACE2 and increase the fusion of spike protein and ACE2 receptor.^[1]^ The conversion of angiotensin II to angiotensin is decreased when the spike protein of virus binds with the ACE2 receptors.^[7,27]^ This high angiotensin II expression are sign of vascular injury and proinflammation associated with neurodegeneration.^[7]^ Cardiovascular functions are associated with ACE2 receptor, which controls blood pressure and vasoconstriction. In brain, ACE2 receptor is expressed in brain stem nucleus and regulate cardio-respiration.^[12,28]^ When spike protein bind with ACE2 receptor in the brain stem, respiration problems are seen in COVID-19 patients.^[12,29]^

### 5.2. Neuronal route

The retrograde and anterograde transport of virus through afferent and efferent nerve ends with the help of motor proteins like kinesins and dynein defined the neuronal route.^[11,20]^ Respiratory, olfactory and enteric nerves are used for the retrograde axonal transport of the virus into the brain. As olfactory bulbs are not protected by BBB and dura, it can easily infect by the SARS-CoV-2 and then directly infect the brain and cause inflammation and demyelination in various parts of the brain such as brain stem, thalamus.^[12,30]^ Nasal passage of virus into the brain causes loss of smell and taste. This route occurs from lung via sensory nerve endings of vagus nerve.^[11,29]^ SARS-CoV-2 also infect the gastrointestinal system and enter brain through sympathetic and enteric nerve.^[11,31]^ The invasion of virus into the brain are restricted by removing the olfactory bulb and nerve in the mice.^[30]^ But the research is going on for the human nervous system.

## 6. Immune mediated infection and inflammatory response

One principal cause of death in COVID-19 patients is acute respiratory distress syndrome (ARDS) which is mainly caused by cytokine storm. Cytokine storm is a severe inflammatory response in various system of body caused by release of many cytokines and chemokines like interleukin (IL)-1beta, IL-10, IL-7, IL-6, IL-2, interferon gamma, tumor necrosis factor alpha (TNF-alpha), macrophage inflammatory protein 1A, IL1 receptor accessory protein, interferon gamma induced protein and GCSF,^[32]^ which can damage multiple organs of human body. A recent study proved that when SARS-CoV-2 infect the CNS, it induces the immune cells of brain like microglia cells and astrocytes, as a result large amount of INF- alpha, IL-1beta, IL-15, IL-12 and IL-6 are released inside the brain^[33]^ that lead to severe CNS damage.^[6,29]^ There is a decrease in the number of CD4 + T cells, CD8 + T cells and lymphocytes, which are symptoms of severity in COVID-19 patients.^[6,34]^ The remaining CD4 + T cells activate the macrophages to produce IL-6.

The overproduction of cytokines, mainly IL-6 cause cytokine storm syndrome, which is very fatal and leads to damage many organs.^[11,33]^ The severity of COVID- 19 symptoms is positively correlated with increase in the IL-6 cytokines,^[35]^ a potent pro-inflammatory substance responsible for activation of immune cells and injury inside the brain. It is predicted that a high mortality rate in the hospitalized patients would be the result of high inflammation all over the body due to cytokine storm,^[25,36]^ diagnosed by high count of platelets and neutrophils and is associated with various PNS manifestations and act as a biomarker for any neurological disease inducing stroke. Low level of effector T cells and high IgG makes a patient immunocompetent, as a result the virus spread uncontrollably causing severe complications.^[25]^ The clinical status of 5 critically ill patients with COVID-19 and ARDS are improved by neutralizing the antibodies in the plasma of recovered patients.^[6,37]^ Cross reaction between antigens and antibodies against SARS-CoV- 2 leads to severe disease like GBS.^[25,38]^ The distribution of SARS-CoV-2 all over the body results from immunosuppression of patients and hyperinflammation results from activation of the immune system inside the patient’s body. Both these conditions cause severe damage. If the virus invades the brain, it is very difficult to remove.^[25]^ Furthermore, in case of SARS-CoV-2 infection, abnormal immune system can cause post infectious disease like GBS, Amish nemaline myopathy, ADEM.^[6]^ In this way, the viral infection can activate the immune system in brain and activated immune cells may cause neurological damage and inflammation.^[6]^

## 7. Coagulopathy and hypoxic injury

This abnormal coagulopathy in COVID -19 patients result from increased in the level of degradation products of fibrin, D dimer and cause long prothrombin and thromboplastin time.^[6,39]^ Moreover, intravascular bleeding also cause death.^[6,40]^ Abnormalities in blood clotting and migration of blood clot into brain arteries leads to stroke.^[12]^ ARDS may also develop which show low oxygen level and breathing shortness.^[6,32]^ Patients with ARDS show pulmonary edema, causes respiratory disorders and hypoxia in the CNS.^[6,34]^ Subsequently, it induces a blockage of cerebral blood flow by accumulating anaerobic metabolites in the mitochondria and acid metabolites in the brain.^[6]^ Thus, hypoxia cause severe damage to the nervous system by becoming a high- risk factor for ischemic stroke and hypoxic encephalopathy. However, the BBB and CSF in the brain prevent the virus from affecting it, still recent studies proved the presence of viral RNA and protein inside the brain and appearance of various neurological complications associated with SARS-CoV-2.^[9,11]^ Once the virus reaches into the CNS, it can bind with its receptor on different parts of CNS and start their replication, which leads to severe infection and alter the cellular processes for protein folding and energy production.^[12,28]^ Besides, the virus induces intracellular protein aggregation or misfolding, increase the reactive oxygen species that may cause lysosome damage and mitochondrial abnormality, ultimately leads to cell death.^[7]^

## 8. Neurological manifestations of COVID-19

**Figure 3.**
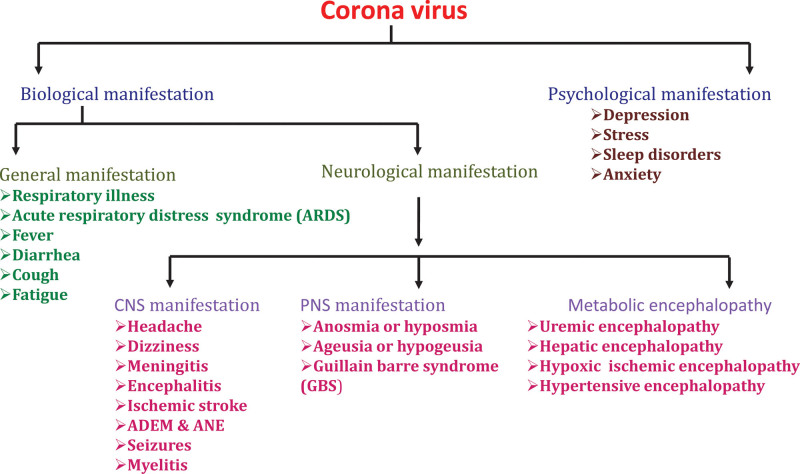
Schematic representation of neurological manifestations of COVID-19. The coronavirus causes various biological and psychological complications. Biological manifestations also include the neurological manifestations that is divided into the CNS manifestations, PNS manifestations and metabolic encephalopathy. CNS = central nervous system, PNS = peripheral nervous system.

As shown in the schematic representation of neurological manifestations of COVID-19 (Fig. 3), various neurological complications are seen in the patients with COVID- 19, which were increasing day by day as COVID-19 disease progress. These neurological manifestations are developed by the direct effect of the virus on nervous system or immune mediated disease after infection. After much research, the neurological complications were divided into 3 parts, CNS manifestations, which include seizure, ataxia, cerebrovascular disease, impaired consciousness, dizziness and headache, PNS manifestations, which include nerve pain, loss of sense of smell and taste, GBS and the third skeletal muscle manifestations.

According to their respiratory status, out of 214 patients, 88 (41.1%) had severe complications and 126 (58.9%) were mildly infected. 78 patients (36.4%) developed neurological disease, among which 53 (24.8%) shows CNS injury, 19 (8.9%) show PNS injury and 23 (10.7%) developed skeletal muscle injury.^[9]^ In 1 national registry of 125 covid-19 patients, psychiatric and neurologic disease were developed over 3-week period. Among those 125 patients, 39 (31%) had changed mental status among which 16(13%) had encephalopathy and 23(18%) were developed neuropsychiatric disease {6 (5%) with neurocognitive syndrome, 4 (3%) with an affective disease and 10 (8%) developed psychosis}. Among 125 patients, 9 (7%) had intracerebral hemorrhage, 57 (46%) had ischemic stroke, 77 (62%) patients developed cerebrovascular disease, 10 (8%) had other cerebrovascular disease and 1 (<1%) developed CNS vasculitis.^[4]^ A case of young females who lost her sense of smell was reported by Eliezer et al, in which her CT scan and MRI report suggest that she had developed inflammation only in the olfactory clefts without any abnormalities in the olfactory tract and bulb.^[41]^ An observational study was done by Li et al, on 221 covid-19 patients and found that 1 (0.5%) developed cerebral hemorrhage, another 1 (0.5%) developed cerebral venous sinus thrombosis and 11 (5%) were developed acute ischemic stroke.^[29]^ The neurological symptoms of the covid-19 patients are not severe, including anosmia and ageusia, headache, dizziness, loss of smell and taste, and malaise.^[9]^ But some patients showing high mortality rate.

## 9. CNS manifestations

The CNS-related manifestations include headache and dizziness, meningitis and encephalitis, encephalopathy, acute cerebrovascular disease and ischemic stroke, ADEM and ANE, seizure and myelitis.

### 9.1. Headache and dizziness

Headache and dizziness are the most observable minor signs shown by 40% of the COVID- 19 patients.^[2,9,42]^ Although headache is a nonspecific symptom of any viral infections, it has been largely discovered worldwide ranging from 3% to 13% and dizziness is discovered among 16% of the patients.^[9,43,44]^ It is due to the decrease function of ACE2 and increased blood pressure in COVID-19 patients. Other reasons are hypoxia and cytokine storm which is developed by increase in the level of cytokines and chemokines, mainly IL-6 and TNF-alpha.^[3,45]^

### 9.2. Meningitis and encephalitis

Meningitis is the inflammation of the meninges and encephalitis is the inflammation of the brain parenchyma, caused by viral infections.^[6]^ Viral encephalitis is characterized by common symptoms like fever, vomiting, headache, convulsion and unconsciousness.^[25,45]^ Early diagnosis of viral encephalitis is very critical. By genome sequencing, the presence of SARS-CoV-2 in the CSF of COVID-19 patients confirmed with the viral encephalitis and it is caused by CoV.^[6,10,11,25,32]^ A case of rhombencephalitis was also reported from COVID-19 patients.^[46,47]^ It is observed that the patient who have meningitis or encephalitis are tested COVID-19 positive because SARS-CoV-2 RNA and proteins are found in their brain tissue and CSF. This report suggested that these complications are caused by the virus itself by infecting and damaging the brain directly.

### 9.3. Encephalopathy

Severe COVID-19 patients develop encephalopathy over hours to days with some common symptoms like systemic inflammation, sepsis, hypoxia, cytokine storm and renal failure which is diagnosed by increase in the level of IL-2, IL-7, GCSF, IL-6, and TNF-alpha1^[48]^ with no evidence of inflammation on CSF analysis.^[45]^ IT was reported in 50% of hospitalized COVID-19 patients. A study of several patients reveals that many patients who died of COVID-19 show hypoxic encephalopathy.^[6,49]^ A 74-years-old man with a past medical history of Parkinson’s disease, atrial fibrillation, chronic obstruction, pulmonary disease and stroke-developed symptoms of encephalopathy like altered mental status and headache and was tested positive for COVID-19.^[6,50]^ The risk of encephalopathy is soaring in case of COVID-19.^[3,50]^ Several types of encephalopathy like hepatic encephalopathy, uremic encephalopathy, hypoxic ischemic encephalopathy, hypertensive encephalopathy are seen in COVID-19 patients.

### 9.4. Uremic encephalopathy

Renal failure along with fall in the glomerular filtration rate is reported in COVID-19 patients results in uremic encephalopathy, which develop symptoms like seizure and fatigue. It also disturbed the ACE/ACE2 ratio.^[3]^

### 9.5. Hepatic encephalopathy

SARS-CoV-2 directly and indirectly affect the liver, causes hepatic disorders.^[3,39]^ Liver injury and buildup of fat inside it was reported in SARS-CoV-2 patients.^[3,34,49]^ Besides, cardiovascular disorders also lead to hepatic encephalopathy.^[3]^

### 9.6. Hypoxic ischemic encephalopathy

It is a neurometabolic and neurovascular disease, which appear due to change in cerebral metabolism and lack of glucose and oxygen supply and influenced the cerebral regions like cerebellum, thalamus, basal ganglia and hippocampal formation.^[3]^ The lack of oxygen or hypoxia condition is developed due to respiration abnormalities, as a result anaerobic metabolism occurs in the mitochondria of cerebral cells. Obstruction in cerebral blood flow and ischemia, vasodilation and brain edema developed because of low pH.^[3,45]^

### 9.7. Hypertensive encephalopathy

The hypertensive encephalopathy patients develop symptoms like headache and seizure. The breakdown of BBB and decrease in the expression of ang II leads to increase in BP, hyperfusion and brain edema, which provoke hypertensive encephalopathy. Comorbidity patients with prior neurological symptoms have a higher risk of developing encephalopathy after COVID-19 infection.^[3,51]^

### 9.8. Acute cerebrovascular disease and ischemic stroke

Cerebrovascular disease, ischemic stroke are important manifestations associated with severely ill COVID-19 patients.^[6,9,52,53]^ Patients under the age of 50 with no history of cerebrovascular disease also suffered from large vessel stroke.^[11,54]^ According to many studies, older patients and comorbidities like hypertension, prior stroke and diabetes are more prone to COVID-19 related stroke and cerebrovascular diseases.^[44]^

ACE2 is responsible for the conversion of ang II, a Powerful Vasoconstrictor to ang (1- 7). But in COVID-19 patients the concentration of ang II increase due to decrease in ACE2 expression after binding of the spike protein of CoV with its receptor. As a result, the risk of development of blood coagulation, severe tissue damage and hypertension increases that ultimately leads to ischemic stroke.^[11,25,55]^ After ischemic stroke, the activated immune cells of brain produce pro-inflammatory mediators that further cause brain tissue injury.^[25,56]^ Another potential cause of neurological damage and stroke in COVID-19 patients are cytokine storm.^[11,49]^ Several studies predict that the cause of mortality in COVID-19 patients with ischemic stroke is due to increase in the neutrophil to lymphocyte ratio, serum ferritin and C- reactive protein.^[25,29,57]^ The increase in neutrophils cause overproduction of neutrophil extracellular traps, which leads to thrombosis. Thrombosis is further increased by SARS-CoV-2 induce damaged endothelial cells leads to decrease in nitric oxide synthase, causing nitric oxide deficiency. As nitric oxide inhibits the adherence of platelets and leukocytes to the endothelium and is a vasodilator, its deficiency enhances the development of stroke.^[25]^

### 9.9. ADEM and ANE

ADEM is a post viral demyelinating disease of unknown cause and mechanism, and is more common in children, also occur in any stage of life after a week of infection.^[6]^ Multifocal neurological symptoms suggest the development of this disease. MRI report of a 51-year-old woman with COVID-19 show ADEM with severe demyelinating symptoms.^[25,58]^ It has also been reported in a 15-year-old boy after the infection of HCoV-OC43, which presence was detected in the CSF and in nasopharyngeal secretion by RT-PCR test.^[2]^ The first case of ANE with COVID-19 was reported by Poyiadji et al, from USA. Here, cerebellum, cerebral white matter and brain stem are affected.^[59,60]^ SARS-CoV-2 infection causes severe cytokine storm that breaks the BBB and cause brain necrosis and neuroinflammation, leads to brain abnormalities.^[25]^

### 9.10. Seizure

Seizure is a baleful complication occur in case of COVID-19 and can develop mainly because of encephalopathy and encephalitis.^[44,61]^ COVID-19 patients with unconsciousness and convulsion showed seizure and encephalitis.^[1,10]^ The main cause and origin of seizure is hypoxemia, decrease expression of ACE2 receptor after binding with spike proteins and increased ang II concentration.^[44,61]^ More cytokine likes IL-1Beta, TNF-Alpha etc. are produced in these conditions that increase the pathogenesis of this virus.^[3,62]^ It is also suggesting that the seizure would be the initial symptoms of COVID-19 patients.^[25,63]^

### 9.11. Myelitis

Along with brain damage, SARS-CoV-2 also infect the spinal cord and cause myelitis.^[6]^ A 66-year-old male COVID-19 patient from Wuhan city had fever and body ache was diagnosed with myelitis and gradually developed lower limb weakness, urinary and bowel incontinence.^[23,60]^ The virus binds with its receptor present on spinal cord neurons and enter it.^[23,44]^ These patients were treated with antibiotics and antiviral substance like intravenous immunoglobulin and steroids and the outcome were also good. Many researchers also suggested that the acute myelitis is also developed due to cytokine storm and over reactive inflammatory response which was confirmed or diagnosed by high concentration of IL-6, serum amyloid-A, C-reactive protein, and serum ferritin.^[60]^ After recovering from COVID-19, lower limb weakness, bladder abnormalities, urinary and bowel incontinence, acute myelitis was developed.^[6,23]^

## 10. PNS manifestations

PNS related manifestations after COVID-19 infections are anosmia or hyposmia, ageusia or hypogeusia and GBS.

### 10.1. Hyposmia and Hypogeusia

The loss of sense of smell is called as anosmia or hyposmia and loss of sense of taste is called hypogeusia or ageusia have reported in COVID-19 patients in many countries worldwide. Many causative viruses, which infect the upper respiratory tract damage the olfactory epithelium, which leads to hyposmia in infected patients.^[2]^ About 30% to 66% develop an early symptom of hyposmia after viral infection, which later on developed hypogeusia.^[2,22,43]^ Initial stages of COVID-19 infection are diagnosed with hyposmia and hypogeusia.^[6,64,65]^

Hyposmia and hypogeusia are more found in COVID-19-positive cases compared to negative cases of COVID-19 of USA. The loss of smell was found in 68% COVID-19 patients while 16% COVID-19 negative patients and loss of taste was found in 71% covid-19-positive patients while only 17% was found without COVID-19.^[60,66]^ The olfactory epithelium express both ACE2 and TMPRS2 receptor, which act as entry site and direct invasion of virus.^[67]^ As a result, the high concentration of ang II destroyed the neural stem cells by apoptosis.^[3]^ Thereby, it reduces the replacement of new neurons in the olfactory bulb and ultimately leads to loss of sense of smell and taste.^[3]^ Here, the virus enters through nasal route.^[60,66]^

### 10.2. GBS

An autoimmune induced nerve disease, which mainly damage the PNS is Guillain barre syndrome. Also called as acute inflammatory demyelinating polyneuritis caused by viral infection or autoimmune response.^[6]^ It can also develop after Epstein Barr virus, cytomegalovirus and campylobacter jejune infection.^[25]^ Several cases of GBS in association with COVID-19 are reported with symptom’s weakness in lower limbs, paraesthesia and tetra paresis. Polyneuritis cranialis and Miller Fisher syndrome are 2 variants of GBS, which have also been developed in COVID-19 patients.^[12,23,25,60]^ The best management of GBS is achieved by intravenous immunoglobulin treatment.^[25,68,69]^

### 10.3. Skeletal muscle manifestations

Skeletal muscle-related complications or myopathy occur in both mildly and severely affected COVID-19 patients. They develop symptoms like fatigue, weakness, myalgia, increase serum creatine kinase and lactate dehydrogenase. The exact mechanism by which SARS-CoV-2 cause skeletal muscle infection is unclear, it occurs directly by binding with ACE2 receptor in the cells of skeletal muscle or due to cytokine mediated inflammation (Cytokine storm).^[25,44,60]^

## 11. Psychological effects of COVID-19

The rapid increase in COVID-19 created a highly panic and wide spread anxiety among individuals, every institution and even governments worldwide. Social distancing, quarantine, mandatory face covering using mask, restricts people from going outside their home, shutting boarders and all over lockdown/shutdown in large cities for long period played major role to prevent the spreading of this disease, but on the other hand it affected people psychologically, economically and sociologically. As a result, mental health issues like depression, stress, sleep disorders and anxiety develop among non-medical and medical person, old age people, children and among overall general population. Other reasons of this situation are loss of income, fear of contracting infection and loss of near and dear ones. According to Lai et al,^[70,71]^ women and nurses are more develop these mental health illnesses. A psychological professor, Steven Taylor claims that, “Coronaviruses pandemic leave psychological scars on people all over the world” and “many people won’t be going back to normal anytime soon.” If this situation continues, it develops psychological effect on the population throughout life.^[44,70,71]^

## 12. Possible therapeutics to prevent COVID-19 infection

ACE2 receptor is expressed in many organs all over the body. By preventing the binding of the spike proteins of SARS-CoV-2 with these ACE2 receptors prevents the damage of these organs. We already know that high tendency of clot formation is seen in severely affected COVID-19 patients. So, the administration of anticoagulant decreases the mortality in COVID-19 patients with coagulopathy. To prevent inflammation in COVID-19 patients, monoclonal antibodies that target IL-6 receptors and plasma therapy are used to improve patient’s conditions. IL-6 receptors are blocked by tocilizumab, and IL-1 receptors are blocked by anakinra to prevent damaged caused by cytokines.^[11]^

## 13. Conclusion

Respiratory and cardiovascular systems are the primary target of SARS-CoV-2, however, if not detected and treated in early phase, it can invade the nervous system and cause severe neurological complications. The present review briefly described the plausible routes and mechanisms of nervous system infection of COVID-19 and the range of neurological manifestations. However, further many research must be required for better understanding the pathogenic mechanism and treatment options of COVID in the nearby future.

## Author contributions

**Conceptualization:** Norazlina Mat Nawi, Santosh Singh.

**Collection literature:** Nikita Meher, Arifullah Mohammed, Mohammad Khairul Azhar Abdul Razab, LVKS Bhaskar, Norazlina Mat Nawi, Santosh Singh.

**Data Analysis:** Nikita Meher, Arifullah Mohammed, Mohammad Khairul Azhar Abdul Razab, LVKS Bhaskar, Norazlina Mat Nawi, Santosh Singh.

**Writing – original draft:** Nikita Meher.

**Writing – review & editing:** Nikita Meher, Arifullah Mohammed, Mohammad Khairul Azhar Abdul Razab, LVKS Bhaskar, Norazlina Mat Nawi, Santosh Singh.
